# Effect of two cosmetic compounds on the growth, biofilm formation activity, and surface properties of acneic strains of *Cutibacterium acnes* and *Staphylococcus aureus*

**DOI:** 10.1002/mbo3.659

**Published:** 2018-06-17

**Authors:** Andrei V. Gannesen, Valerie Borrel, Luc Lefeuvre, Alexander I. Netrusov, Vladimir K. Plakunov, Marc G. J. Feuilloley

**Affiliations:** ^1^ Department of Microbiology Faculty of biology Lomonosov Moscow State University Moscow Russia; ^2^ Laboratory of petroleum microbiology Winogradsky Institute of Microbiology Research Center of Biotechnology Russian Academy of Sciences Moscow Russia; ^3^ Laboratory of Microbiology Signals and Microenvironment LMSM EA4312 University of Rouen Normandy, Normandie Université Evreux France; ^4^ Uriage Dermatological Laboratories Neuilly‐sur‐Seine France

**Keywords:** biofilm, cosmetics, *Cutibacterium acnes*, metabolism, *Staphylococcus aureus*, surface adhesion, surface polarity

## Abstract

Increasing popularity of preservative‐free cosmetics necessitates in‐depth research, specifically as bacteria can react to local factors by important metabolic changes. In this respect, investigating the effect of cosmetic preparations on pathogenic strains of commensal species such as acneic forms of *Cutibacterium acnes* (former *Propionibacterium acnes*) and bacteria behaving both as commensals and opportunistic pathogens such as *Staphylococcus aureus* is of major interest. In this study, we studied the effect of commonly used cosmetics, Uriage^™^ thermal water (UTW) and a rhamnose‐rich polysaccharide (PS291^®^) on RT4 and RT5 acneic strains of *C. acnes* and a cutaneous strain of *S. aureus*. UTW affected the growth kinetic of acneic *C. acnes* essentially by increasing its generation time and reducing its biomass, whereas only the *S. aureus* final biomass was decreased. PS291 had more marginal effects. Both compounds showed a marked antibiofilm activity on *C. acnes* and *S. aureus*. For *S. aureus* that appeared essentially due to inhibition of initial adhesion. Cosmetics did not modify the metabolic activity of bacteria. Both *C. acnes* and *S. aureus* showed marked hydrophobic surface properties. UTW and PS291 had limited effect on *C. acnes* but increased the hydrophobic character of *S. aureus*. This work underlines the effect of cosmetics on cutaneous bacteria and the potential limitations of preservative‐free products.

## 1 INTRODUCTION

Human skin is the largest human organ, which harbors hundreds of microbial genera (Grice et al., 2009). This coexistence developed in the course of evolution: while the host organism does not prevent growth of commensal microorganisms, it attacks pathogenic invaders (sometimes with the aid of commensals) (Chiller, Selkin, & Murakawa, 2001). Species ratios of microorganisms on human skin depends on sex, age, geographic region, etc., although the patterns within a group are almost the same (Grice et al., 2009; San Miguel & Grice, 2014). Microenvironment determined by the physiological properties of skin is among the most important factors affecting microbial growth. Three types of physiological environment, greasy, moist, and dry, may be discerned depending on position of a skin sample and localization of sweat and oil glands (Grice et al., 2009). Each type is characterized by different ratios of microbial species. Actinobacteria (particularly *Cutibacteria*) and Firmicutes (essentially *Staphylococci*) prevail at greasy and moist sites, while Betaproteobacteria and Corynebacteria predominate at dry ones (Grice et al., 2009; San Miguel & Grice, 2014).

*Cutibacterium acnes* (former *Propionibacterium acnes*) (Scholz & Kilian, 2016) is the most common member of the genus associated to the human skins (Bruggemann, 2004). These are gram‐positive diphtheroid aerotolerant rod‐shaped anaerobic microorganisms (Aubin, Portillo, Trampuz, & Corvec, 2014). *C. acnes* is a component of the normal microbiota, an opportunistic pathogen preferring anoxic, fat‐rich niches, such as sebaceous glands (Aubin et al., 2014; Bruggemann, 2004; Fitz‐Gibbon et al., 2013). *C. acnes* is traditionally considered involved in the pathogenesis of many cases of acne, one of the most common skin disorders, although direct evidence is still lacking (Achermann, Goldstein, Coenye, & Shirtliff, 2014; Fitz‐Gibbon et al., 2013). In this regard, *C. acnes* biofilms, found inside sebaceous glands, are considered among a possible major causes of acne (Coenye, Peeters, & Nelis, 2007) and, as these biofilms provide high resistance to antimicrobial agents and to the action of the host immune system (Achermann et al., 2014; Coenye et al., 2007; Nikolaev & Plakunov, 2007; Nozhevnikova, Botchkova, & Plakunov, 2015), they play a critical role *C. acnes* in the pathogenicity. *C. acnes* is also a species known for its high heterogeneity and it was divided into four phylogenetic groups (IA, IB, II and III), group IA being associated to the more severe forms of acne (McDowell et al., 2010). More recently, the collection, ribotyping, and genome sequencing of *C. acnes* on normal skin and on the skin of volunteers suffering of diverse degree of acne, revealed that some principal ribotypes bearing a specific locus (Locus 3) of plasmidic origin are associated to acne (Fitz‐Gibbon et al., 2013). Environmental parameters, and particularly cosmetic compounds, should be also modulating the biofilm formation activity and virulence of these microorganisms (Enault, Saguet, Yvergnaux, & Feuilloley, 2014) but until now these studies were never realized on acneic strains of *C. acnes*. In its ecological niche, such as the hair follicle, *C. acnes* is associated to another lipophilic microorganism, *Staphylococcus aureus* (Frenard et al., 2018), a gram‐positive facultative anaerobe belonging to the phylum Firmicutes. *S. aureus* is a component of the normal skin microbiome (Aryee & Edgeworth, 2017) although it can also behave as an opportunistic pathogen and recent studies suggest that *C. acnes* should participate to *S. aureus* skin colonization and biofilm formation (Tyner, Patel, Tyner, & Patel, 2016).

Considering the high heterogeneity of *C. acnes* (McDowell et al., 2010) it appeared essential to fully document the biofilm formation activity and response to cosmetic compounds of the more critical of those bacteria associated to acne, such as RT4 and RT5 strains which bear a virulence plasmid associated to the more severe forms of acne (Fitz‐Gibbon et al., 2013). In the present work we investigated the effect of Uriage^™^ thermal water (UTW) and rhamnose‐rich polysaccharide (PS291^®^). UTW is a natural isotonic mineral solution from and Alpine spring and is often used for face care (Uriage, 2018). PS291, also designated as Teflose for its coating activity (Solabia Biotecnolgica, 2018) is a branched polysaccharide containing rhamnose, glucuronic acid, and glucose. Both compounds enter into the formulation of cosmetic products designed for sensitive skin, mild to moderate acneic skin, as well as in the used in deodorants or antiperspirants. The action of these compounds on *C. acnes* was compared to that on a strain of *S. aureus*, MFP03, collected on human skin exempt of any clinical sign (Hillion et al., 2013).

## 2 MATERIALS AND METHODS

### 2.1 Bacterial strains and culture

*Cutibacterium* (former *Propionibacterium* [4]) *acnes* acneic strains ribotype 4 (RT4) HL045PA1 and ribotype 5 (RT5) HL043PA2 initially isolated by Fitz‐Gibbon (Fitz‐Gibbon et al., 2013) were obtained from BEI Resources American Type Culture Collection (Virginia, United States). These strains associated to severe forms of acne differ from nonacneic strains (ribotype 6 and in a lower extend ribotypes 1, 2, and 3) by a large plasmide (Locus3) which should confer their virulence properties (Fitz‐Gibbon et al., 2013). Bacteria stored at −80°C were initially plated on agar brain heart infusion (BD). As these strains are strictly anaerobic, the plates were incubated for 72 hr in BD GasPack^™^ under anoxic conditions at 37°C. Colonies were then transferred into sterile conical 15 ml tubes (Falcon) filled to maximal capacity with reinforced clostridial medium (RCM) and grown for 72 hr at 37°C. For all experiments *C. acnes* was grown in RCM.

*S. aureus* MFP03 was initially isolated from the skin of healthy volunteers (Hillion et al., 2013). It was characterized by phenotypic, metabolic, 16sRNA sequencing and MALDI‐biotyper total proteome analysis. For culture, an aliquot of bacteria stored at −80°C (± 0.5 mg) was mixed in 3 ml of the Luria‐Bertani (LB) broth and incubated for 24 hr at 37°C under agitation. For microcolony formation in dynamic conditions and adhesion to solvents *S. aureus* MFP03 was grown in tryptone‐soy broth (Biocar Diagnostics) with 0.25% glucose.

### 2.2 Tested compounds

UTW is a natural isotonic water (pH ≈ 7.0) containing approximately 11 g/L salts. Its mineral composition (g/L) is: sulfates, 2.86; chlorides, 3.5; and bicarbonates, 0.39. Elemental composition is as follows: sodium, 3.5 g/L; calcium, 0.6 g/L; magnesium, 0.125 g/L; potassium, 0.0455 g/L; silicon, 0.042 g/L; zinc, 0.16 mg/L; manganese, 0.154 mg/L; copper, 0.075 mg/L; and iron, 0.0,015 mg/L The water is stored in sterile conditions at room temperature. Controls were realized by use of physiological water (NaCl 0.9% pH = 7.0—PS). PS291^®^ is a rhamnose‐rich polysaccharide obtained by fermentation and patented by BioEurope. It was used as a saturated solution in a water‐propanediol mixture. These compounds were selected in coherence with previous studies labeled by the French national cluster “Cosmetic Valley” (Enault et al., 2014; Mijouin et al., 2013) and CNRS GDR Cosm'Actif. ETU and PS291 were tested at 50% and 4%, respectively, corresponding to the concentrations potentially present on skin during exposure to cosmetics. As described in the result chapter, the effect of ETU on bacteria was compared to that observed using a same percentage of PS or complete RCM. For studies realized on PS291 the control medium was supplemented with a same amount of water‐propanediol mixture.

### 2.3 Bacterial growth kinetic

Effect of UTW and PS291 on the growth of *C. acnes* and *S. aureus* was studied in the F system SAFAS spectrophotometer incubators (Flex‐Xenius XM; SAFAS, Monaco). The SAFAS system uses 96‐well flat‐bottomed polystyrene plates (NUNC). The inoculum was added to an initial OD_580_ = 0.08–0.1. In the case of *C. acnes*, peripheral wells were filled with a CO_2_‐producing solution and prepared in anoxic conditions using GasPack^™^ system and sealed with Parafilm before incubation. Bacteria were incubated at 37°C for 72 hr (*C. acnes*) or 24 hr (*S. aureus*). Optical density of the cultures was determined automatically every 15 min. Growth curves were determined over a minimum of three independent experiments. Statistical variability was minimal and error bars (*SEM*) do not appear in the figure.

### 2.4 Biofilm investigation using crystal violet staining

Biofilms were grown in NUNC. Experiments were carried out according to the modified classical procedure (O'Toole, 2011). The mixture (200 μl) of the medium and solutions of tested compounds was dispensed into the wells, and inoculum with OD_580_ = 1.7 ± 0.2 was added. The ratio of inoculum and medium volumes for *S. aureus* and *C. acnes* were 1:40 and 1:6, respectively. The plates were incubated at 37°C: 24 hr on the shaker for *S. aureus* and under static conditions in a GasPack^™^ system for 72 hr for *C. acnes*. Planktonic cultures were then removed, and the wells were washed thrice with PS to remove remaining planktonic cells. The plates were then dried for 5–10 min at 60°C, and the biofilms were fixed with 96% ethanol for 15 min. After fixation, ethanol was removed, the plates were dried and stained with 0.1% crystal violet (CV) for 15 min. After staining, the plates were washed with water for complete removal of dissolved CV. Biofilm‐bound CV was extracted with 96% for 60 min, and optical density of the extract was measured at λ = 595 nm on a Biorad spectrophotometer. Four wells were used for every sample. The experiments were carried out in at least three replicates. The results are expressed as *M* ± *SEM* and statistical differences were determined using the Mann–Whitney nonparametric criterion. Graph plotting and calculation were carried out using Microsoft Excel 2007. The differences between experimental and control variants were considered reliable at confidence coefficient >95% (*p *<* *0.05).

### 2.5 Confocal laser scanning microscopy

Observations were realized using an LSM 710 inverted confocal laser‐scanning microscope (Zeiss, Germany). Three‐dimensional (3D) images and orthocuts were obtained using Zen^®^ 2009 software. Biofilm thickness was quantified with the same software. For these studies bacteria were grown in 24‐well plate with flat glass bottom (Sensoplate, Geiner bio‐one, Germany). Biofilms were grown according to Coenye (Coenye et al., 2007) with modifications. *C. acnes* cultures in RCM (72‐hr old) were collected by centrifugation, washed twice with sterile PS, and resuspended in sterile PS at OD_580_ = 1. The suspension (300 μl) was applied to the wells and incubated for 2 hr at room temperature under anoxic conditions. The suspension was then removed, and the wells were washed twice with sterile PS. One well was fixed for subsequent visualization of primary bacterial adhesion. The medium (1 ml) supplemented with tested substances was then dispensed into the wells. The plates were incubated for 72 hr under static conditions at 37°C. The same technique was used for *S. aureus* except that the culture was realized in normal atmosphere and avec 24°C.

For visualization, the biofilms were stained with SYTO9 Green and subsequently treated with ProLong^®^ Diamond Antifade Mountant (Molecular Probes ^™^). Images are representative of the biofilm structure observed in a mean of 20 different fields over a minimum of four independent studies. Biofilm density and thickness were calculated over the same number of observations using Zeiss Zen Image Analysis software for light microscopy (ImageJ software package). Statistical differences were determined using the Mann–Whitney nonparametric criterion. The differences between experimental and control variants were considered reliable at confidence coefficient >95% (*p *<* *0.05).

### 2.6 Study of microcolony formation in dynamic conditions

In order to differentiate potential effects on the initial adhesion step of *S. aureus*, an original technique of microcolony formation in dynamic conditions was employed. In this case, 1 ml of medium supplemented with the tested substances was dispensed into the wells and inoculated (1:40) with the bacterial strain. No adsorption phase was allowed and the plate was immediately incubated on a shaker at 180 rpm for 24 hr at 37°C. In this condition, only a subset of the more adherent bacteria can interact with the surface and form microcolonies into the wells. These microcolonies were stained with SYTO9 Green, as previously described, and observed using the LSM 710 inverted confocal laser scanning microscope. Images are representative of microstructure observed in a mean of 20 different fields over a minimum of three independent studies. The results are expressed as *M* ± *SEM* and statistical differences were determined using the Mann–Whitney nonparametric criterion. Graphs were calculated using Microsoft Excel 2007. The differences between experimental and control variants were considered reliable at confidence coefficient >95% (*p *<* *0.05).

### 2.7 Investigation of bacterial metabolic activity

The potential effect of UTW and PS291 on the bacterial metabolic activity in biofilms was determined as described by Plakunov (Plakunov, Mart'yanov, Teteneva, & Zhurina, 2016). The method is based on biofilm staining with 3‐(4, 5‐dimethyl‐2‐thiazolyl)‐2, 5‐diphenyl‐2H‐tetrazolium bromide (MTT). MTT is used to reveal viable, metabolically active cells, acting as an electron acceptor for the electron transport chain; as a result, it is converted to water‐insoluble, blue formazan (Berridge & Tan, 1993). The biofilms were grown on GF/F glass fiber filters (Whatman^®^) in Petri dishes with agar medium. *C. acnes* RT4 and RT5 were grown using solid RCM medium. Tryptone‐soy agar with 0.25% glucose was used for *S. aureus* MFP03. Sterile filters (2 × 2 cm) were placed on the agar, and the culture (50 μl) was applied to the center. OD_540_ values for *C. acnes* and *S. aureus* were 1.0 and 0.1, respectively. The biofilms were grown at 37°C for 72 hr (*C. acnes*) or 24 hr (*S. aureus*). After incubation the biofilm‐bearing filters were stained with 0.1% MTT in sterile LB for 20 min, washed with distilled water, and dried. Formazan was then extracted with dimethyl sulfoxide (DMSO, EKOS‐1, Russia; Pariteks, Russia). Optical density of the extract characterizing the number of metabolically active cells in the biofilm was measured at *λ* = 590 nm. All experiments were carried out in at least three replicates. The data were analyzed using the Mann–Whitney nonparametric criterion. The differences between experimental and control variants were considered reliable at confidence coefficient >95% (*p* < 0.05).

### 2.8 Characterization of bacterial surface polarity

Cultures of *C. acnes* and *S. aureus* in stationary phase were harvested (7,000 × *g*; 10 min) and washed twice in PS. The polarity of control and treated bacteria was studied using the Microbial Adhesion to Solvent (MATS) technique (Bellon‐Fontaine, Rault, & van Oss, 1996) and four solvents: chloroform, hexadecane, ethyl acetate, and n‐decane. Briefly, bacterial cultures in early stationary phase and growth in the presence of ETU 50%, PS291^®^ or the respective control medium, were harvested by centrifugation at 10,000 ×* g*. Pellets were rinsed 2 times with NaCl 0.9% in water and diluted to OD_400_ 0.8. An aliquot of bacterial suspension (2.6 ml) at was mixed for 60 s with 0.4 ml of each of the solvents indicated above. The tubes were vigorously hand‐shaken and the OD_400_ of the aqueous phase was measured after 15 min of decantation. In these conditions, bacteria are distributing between the water and solvent phases following their surface hydrophobic or hydrophilic character and Lewis acid/base balance (electronegative or electropositive character). The OD of the aqueous phase was measured at 400 nm. The percentage of cells in each solvent was calculated using the following equation: (1 − *A*/*A*_0_) × 100. The experiments were carried out in at least five replicates. The data were analyzed using the Mann–Whitney nonparametric criterion. The differences between experimental and control variants were considered reliable at confidence coefficient >95% (*p *<* *0.05).

## 3 RESULTS

### 3.1 Effect of thermal water and PS291^®^ on the growth kinetics of acneic strains of *C. acnes* and *S. aureus*

**Figure 1:**
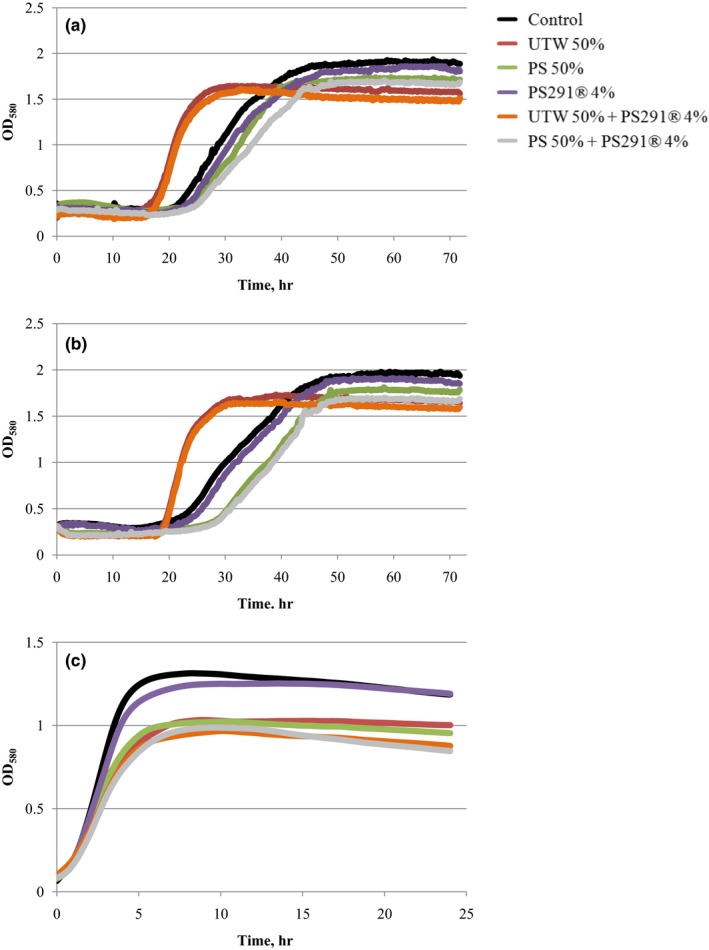
Effect of thermal water and rhamnose‐rich polysaccharide (PS291^®^) on the growth kinetics of acneic strains of *Cutibacterium acnes* and *Staphylococcus aureus*. (a) Effect of Uriage thermal water (UTW) and PS291^®^ on the growth kinetics of acneic strain of *C. acnes* RT4. (b) Effect of UTW and PS291^®^ on the growth kinetics of acneic strain of *C. acnes* RT5. (c) Effect of UTW and PS291^®^ alone or in association on the growth kinetics a cutaneous strain of *S. aureus* (MFP03). Physiological water (PS; NaCl 0.9%) and medium supplement with 4% water‐propanediol mixture were used as controls of UTW and PS291^®^, respectively. Curves are the mean of three independent experiments. Statistical variability was minimal and error bars (*SEM*) do not appear in the figure

Both acneic strains of *C. acnes* had a general similar behavior when they were exposed to UTW or PS291^®^. In the case of the RT4 strain, addition of PS291 was only associated to a minor shift of the growth kinetic but the slope of the curve (generation time) and the plateau (biomass) were almost identical (Figure 1a). In contrast, UTW 50% markedly reduced the lag time of the *C. acnes* growth curve with a 50% ODmax that was reached after only 30 hr in the presence of UTW whereas it required 45 hr in the control study. Conversely the ODmax of the bacterial grown in the presence of ETU was reduced suggesting a decrease in biomass formation. The reaction of the RT5 strain of *C. acnes* to PS291 was different with an apparent increase of the mean generation time from 0500 to 0530 hr, whereas the final OD and biomass was identical (Figure 1b). As observed with the R4 strain, UTW also reduced the lag time (from 44 hr in the control to 29 hr for UTW treated bacteria).

As observed with *C. acnes*, when *S. aureus* MFP03 was exposed to PS291 4% only minor changes in the growth kinetic were observed (Figure 1c). Conversely, UTW 50% induced a marked decrease of the ODmax (*M* = −23%) whereas the generation time appeared unchanged. PS 50% had identical effects with a reduction of the final biomass without noticeable change of the generation time. The behavior of *S. aureus* in the presence of UTW 50% + PS291 4% was identical to that observed using UTW alone. By the same way, PS 50% + PS291 was leading to a reduction of the total biomass identical to that observed using PS 50% alone.

### 3.2 Effect of thermal water and PS291^®^ on the biofilm formation activity of acneic strains of *C. acnes* and *S. aureus*

**Figure 2:**
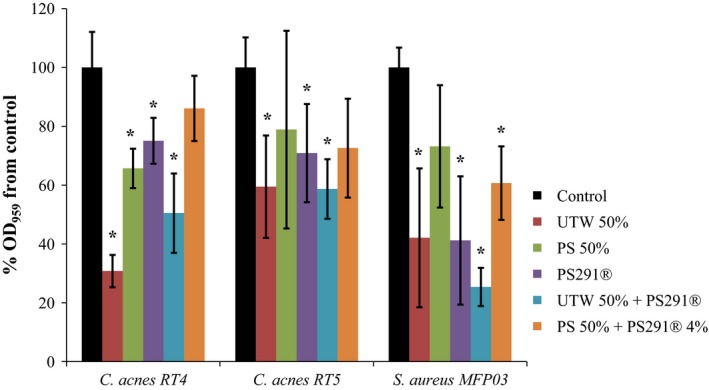
Effect of thermal water and rhamnose‐rich polysaccharide (PS291^®^) on the biofilm formation activity of acneic strains of *Cutibacterium acnes* and *Staphylococcus aureus*. The effect of Uriage thermal water (UTW) and PS291^®^ on biofilm formation activity of acneic strains of *C. acnes* RT4 and 5 and of the cutaneous strain of *S. aureus* (MFP03) was measured by the crystal violet technique. Control studies were realized as indicated in Figure 1. All experiments were carried out in at least three replicates (*p < 0.05)

The study was realized using the crystal violet technique which allows a straining of both bacteria and biofilm extracellular matrix. As shown in Figure 2, UTW 50% and PS291 4% had a strong effect on the biofilm formation activity of *C. acnes* RT4 and RT5 strains that was reduced to 30.8 ± 17.4 and 59.5 ± 23.6% of the control with UTW 50% and 70.9 ± 16.7 and 41.2 ± 21.8% of the control with PS291 4%. PS 50% also reduced the biofilm formation of *C. acnes* RT4 and RT5 to 78.9 ± 33.6 and 65.7 ± 6.7 of the control. As observed in growth kinetic studies, no visible additive effect of UTW with PS291 was observed when bacteria were exposed to the two factors in association. In the presence of UTW 50% and PS291^®^ 4%, the biofilm formation activity of the strains of *C. acnes* RT4, RT5, and *S. aureus* reached 50.5 ± 13.5, 58.7 ± 10.3, and 25.4 ± 6.5% of the control, respectively. However, the inhibitory effect of the association of UTW 50% with PS291 4% was stronger than when PS291 was associated to PS 50%. In this case, the biofilm formation activity of *C. acnes* RT4, RT5, and *S. aureus* reached only 86.1 ± 11.1, 72.6 ± 16.8, and 60.7 ± 12.5 of the control, respectively.

**Figure 3:**
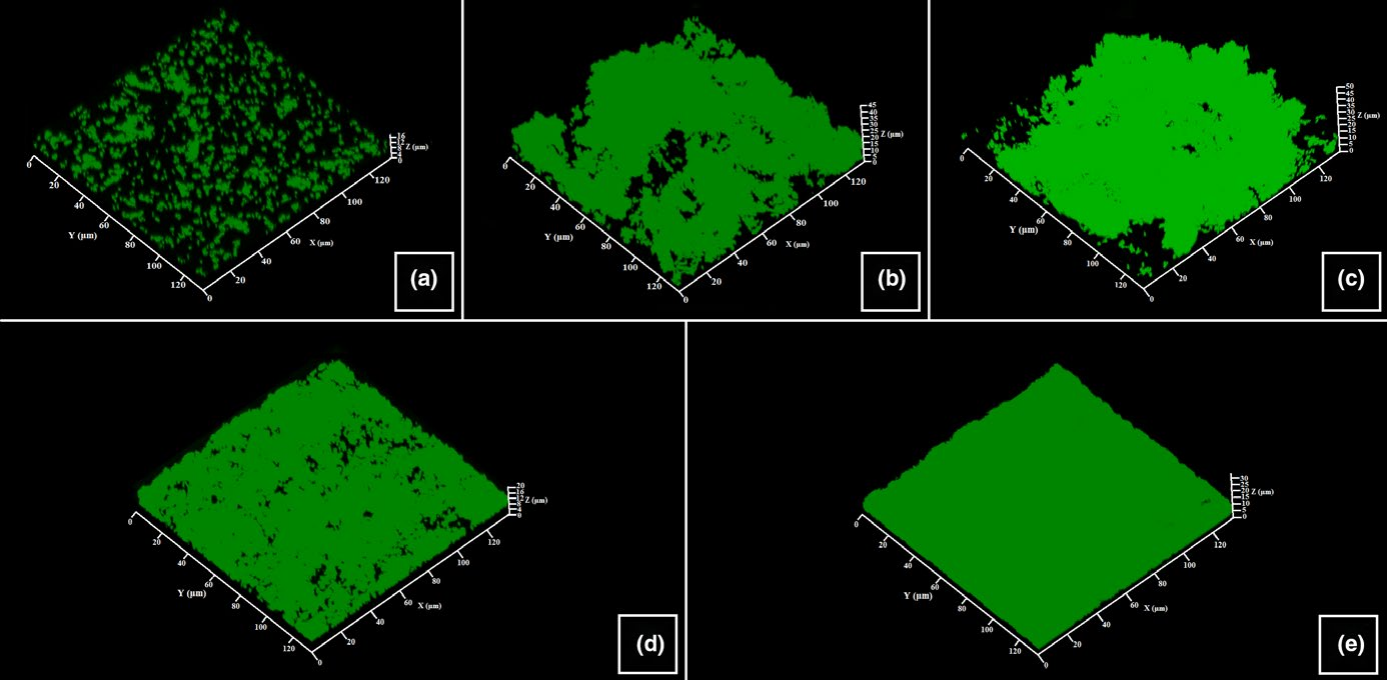
Effect of thermal water and rhamnose‐rich polysaccharide (PS291^®^) on the structure of biofilms formed by the RT4 acneic strain of *Cutibacterium acnes*. Biofilm formation was monitored using Syto 9 green. Horizontal and transverse X/Z views were obtained and submitted to analysis. (a) Control initial cell adhesion after 2 hr. (b) Control biofilm after 72 hr. (c) Biofilm formed in the presence of PS 50%. (d) Biofilm formed in the presence of ETW 50%. (e) Biofilm formed in the presence of PS291 4%. All experiments were carried out in at least three replicates. Control studies were realized as indicated in Figure 1

The structure, thickness, and biomass density of the biofilms formed by *C. acnes* RT4, RT5, and *S. aureus* MFP03 in the presence of UTW and PS291 was investigated by confocal laser scanning microscopy and image analysis. Using the *C. acnes* RT4 we observed a partial biofilm formation with 2 hr that was corresponding to the initial adhesion step (Figure 3a). A mature biofilm (mean thickness = 35.3 ± 6.2 μm/density = 30.3 ± 6.4 μm^3^/μm^2^) was obtained after 72 hr of incubation (Figure 3b). When *C. acnes* RT4 was grown in the presence of PS 50% the mean thickness and biofilm density were not significantly modified (thickness = 32.6 ± 5.1 μm/density = 28.7 ± 4.7 μm^3^/μm^2^) (Figure 3c). UTW 50% had a more visible effect, with a reduction of biofilm thickness and density to 24.9 ± 4.4 μm (−29.4%) and 21.7 ± 4.1 μm^3^/μm^2^ (−28.3%), respectively, (Figure 3d). PS291 4% also decreased the mean biofilm thickness and density (−31.4% and −28.3%, respectively) (Figure 3e). At the opposite of crystal violet observations, the association of UTW and PS291 was leading to a higher decrease in biofilm formation with a reduction of the mean thickness of 40.8% and of density of 35.6%. No major rearrangement of the biofilm organization was observed.

**Figure 4:**
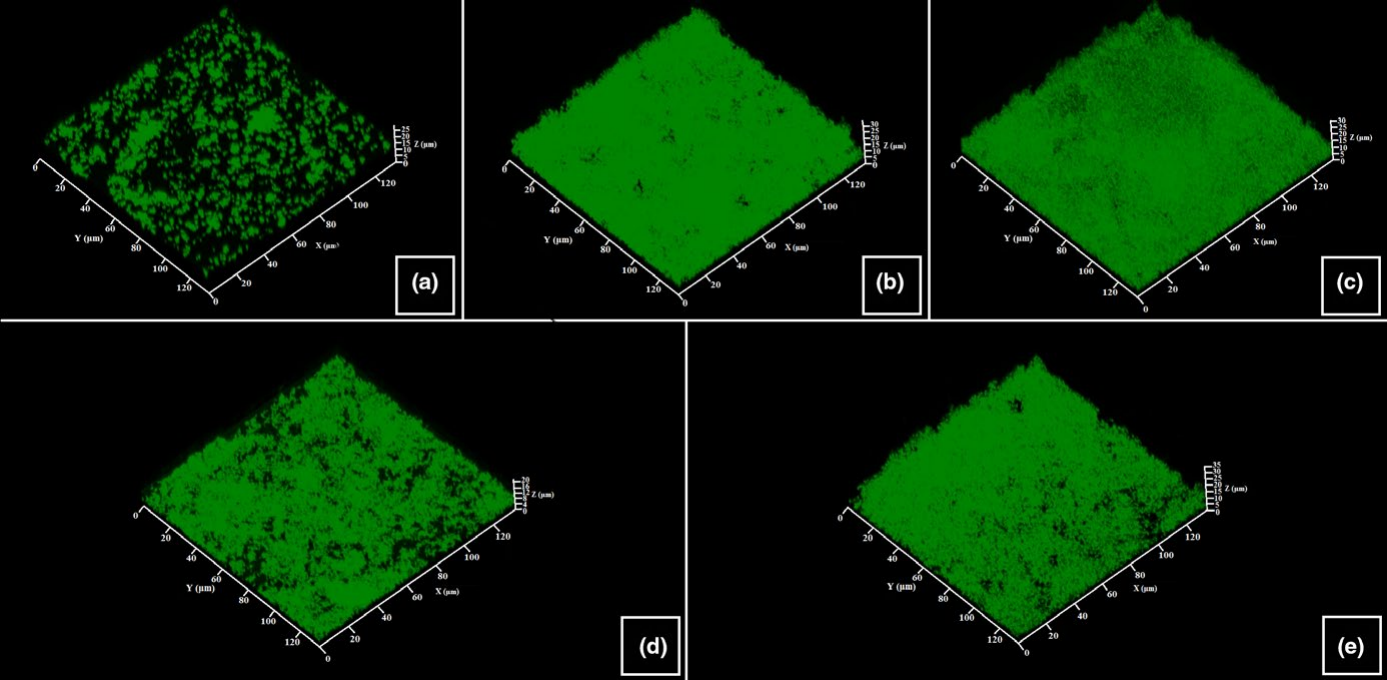
Effect of thermal water and rhamnose‐rich polysaccharide (PS291^®^) on the structure of biofilms formed by the RT5 acneic strain of *Cutibacterium acnes*. Biofilm formation was monitored using Syto 9 green. Horizontal and transverse X/Z views were obtained and submitted to analysis. (a) Control initial cell adhesion after 2 hr. (b) Control biofilm after 72 hr. (c) Biofilm formed in the presence of PS 50%. (d) Biofilm formed in the presence of ETW 50%. (e) Biofilm formed in the presence of PS291 4%. All experiments were carried out in at least three replicates. Control studies were realized as indicated in Figure 1

The reaction of the RT5 strain of *C. acnes* to UTW PS291 alone or in association was close to that of the RT4 strain (Figure 4). PS was also without significant effect on the thickness and density of the biofilms, a marginal increase was even observed (+4.4% and +4.7%, respectively). The inhibitory effect of UTW and PS291 on the biofilm formation was lower than observed on the RT4 strain with a decrease of the mean thickness of only 14.2% with UTW 50% and only 5.3% with PS291 4%. Even the association of UTW with PS291 was less efficient on the formation of these biofilms (−24.4%). As previously noted the structure of the biofilm was not modified.

**Figure 5:**
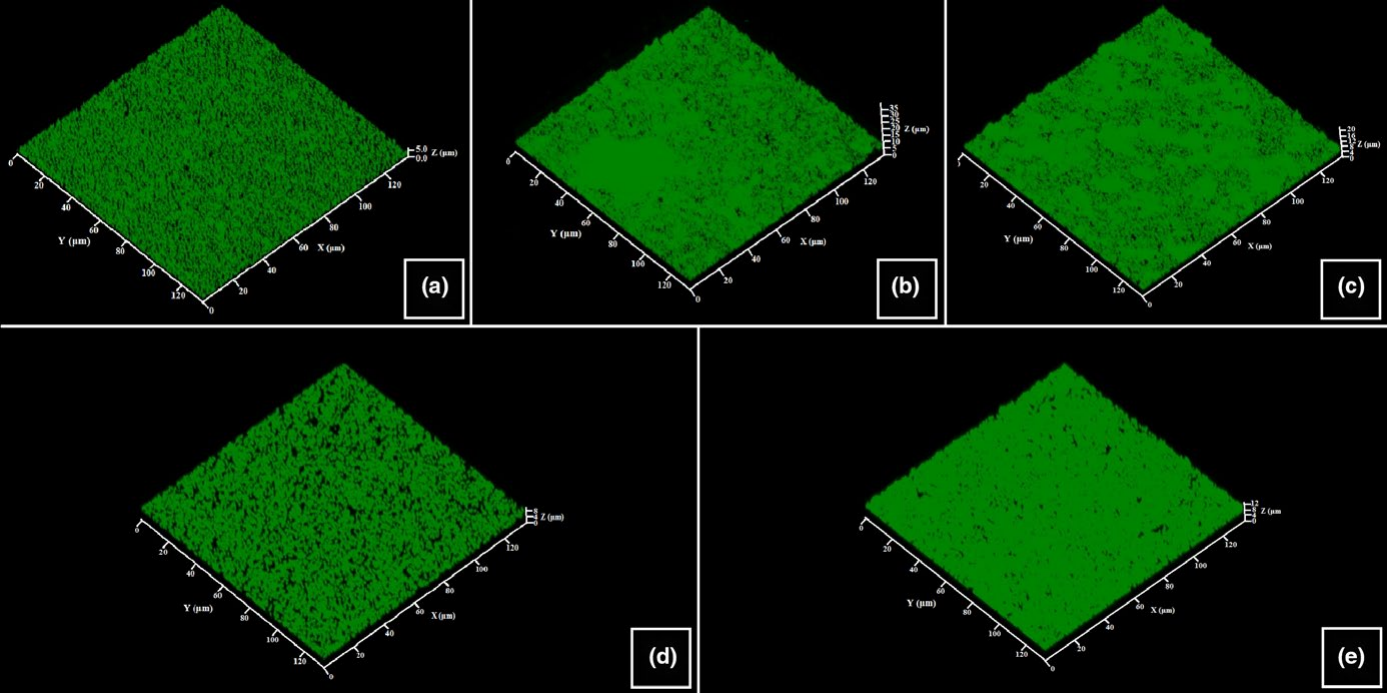
Effect of thermal water and rhamnose‐rich polysaccharide (PS291^®^) on the structure of biofilms formed by the human skin strain *Staphylococcus aureus* MFP03. Biofilm formation was monitored using Syto 9 green. Horizontal and transverse X/Z views were obtained and submitted to analysis. (a) Control initial cell adhesion after 2 hr. (b) Control biofilm after 24 hr. (c) Biofilm formed in the presence of PS 50%. (d) Biofilm formed in the presence of UTW 50%. (e) Biofilm formed in the presence of PS291 4%. All experiments were carried out in at least three replicates. Control studies were realized as indicated in Figure 1

The formation of biofilm by *S. aureus* MFP03 is more rapid than observed wit *C. acnes*. Even after 2 hr of initial adhesion an almost complete bacterial mat was observed (Figure 5a). Mature biofilm was not thick (mean thickness = 16.7 ± 1.5 μm) but occasionally showed mushroom‐like structure of higher dimension (Figure 5b). As observed with the two *C. acnes* strains, PS 50% did not modify the mean thickness and density of *S. aureus* biofilms. UTW and PS291 reduced *S. aureus* biofilm formation both in terms of mean thickness (−20.3% and −23.7%, respectively) and density (−16.1% and −15.2%, respectively), but this decrease was less important than observed using the *C. acnes* strains. Even the association of UTW with PS291 was less efficient than noted with *C. acnes* with a mean reduction of the biofilm thickness of only 20.9% and no obvious additive effect in comparison of UTW alone.

### 3.3 Effect of ETU and PS241 on *S. aureus* microcolony formation in dynamic conditions

**Figure 6:**
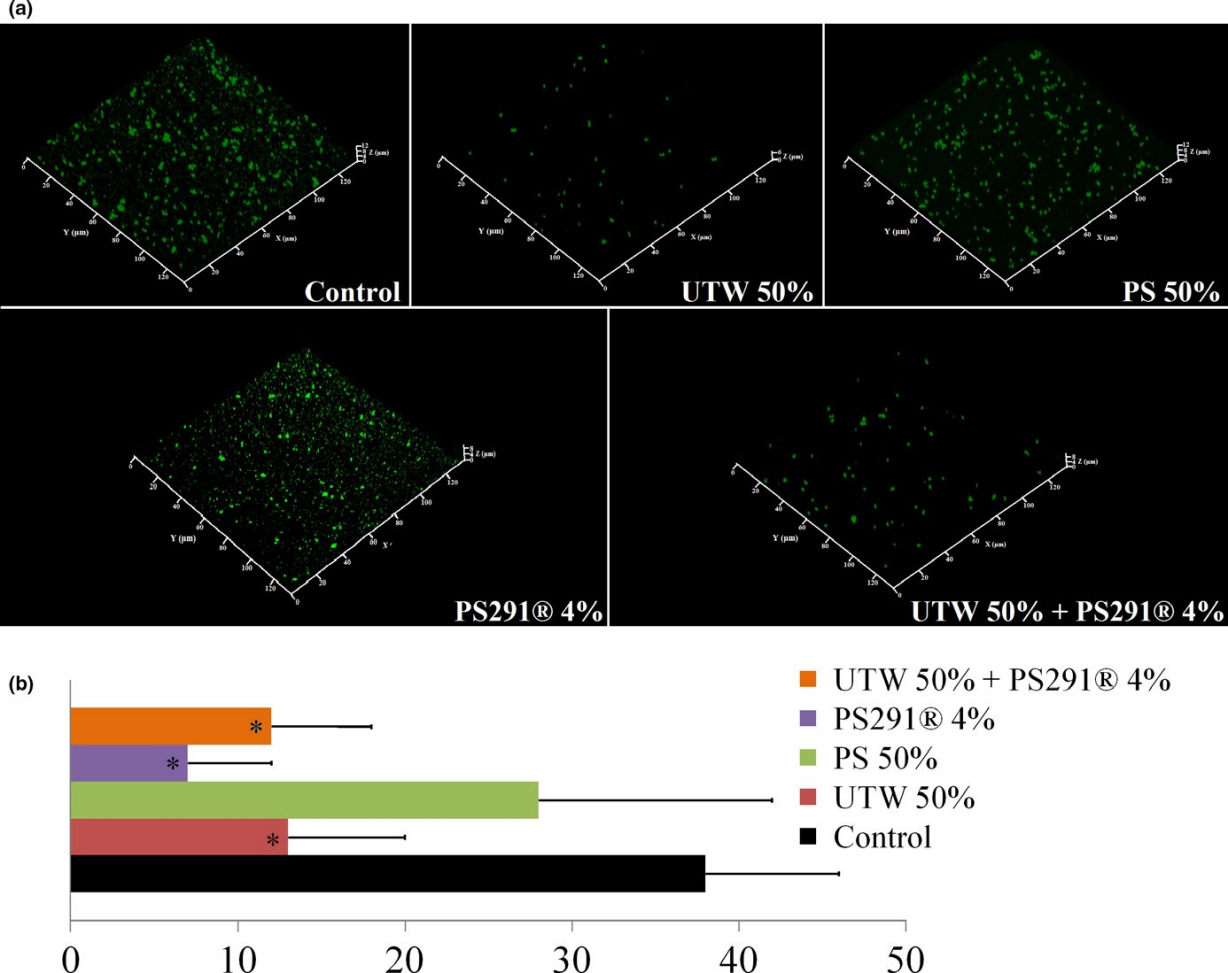
Effect of thermal water and rhamnose‐rich polysaccharide (PS291^®^) on *Staphylococcus aureus* MFP03 microcolonies formation in dynamic conditions. In order investigate the equilibrium between planktonic and adherent bacteria, *S. aureus* MFP03 was incubated in microwells under agitation and microcolonies formation was monitored after 24 hr. (a) Structure of microcolonies formed in the absence of treatment and in the presence of PS 50%, UTW 50%, PS291 4% and UTW 50% + PS291 4%. (b) Mean number of microcolonies formed by *S. aureus*. All experiments were carried out in at least three replicates. Control studies were realized as indicated in Figure 1 (*p < 0.05)

As the initial adhesion step of *S. aureus* was particularly rapid, we used an original approach to investigate a potential effect of UTW and PS291 on the equilibrium between biofilm and planktonic bacteria by growing these cells without preliminary adhesion step and under permanent agitation during 24 hr. This was resulting into the formation of microcolonies on the bottom of the wells (Figure 6a). Cell aggregates with a minimal area of 2.7 μm^2^ were considered microcolonies and counted. The number of microcolonies in the absence of treatment reached a mean of 38/field (14,400 μm^2^) (Figure 6b). Addition of UTW 50% resulted in a decrease of to 13/field. In the presence of PS291 4%, the number of microcolonies decreases to 7/field. Conversely, PS 50% only reduced the number of microcolonies to 28/field. The association of UTW with PS291 was not leading to a additive effect in composition of the effect of the compounds used separately as 12 microcolonies/field were counted in this condition.

### 3.4 Effect of thermal water and PS291^®^ on the metabolic activity of *C. acnes* and *S. aureus* in biofilms

**Figure 7:**
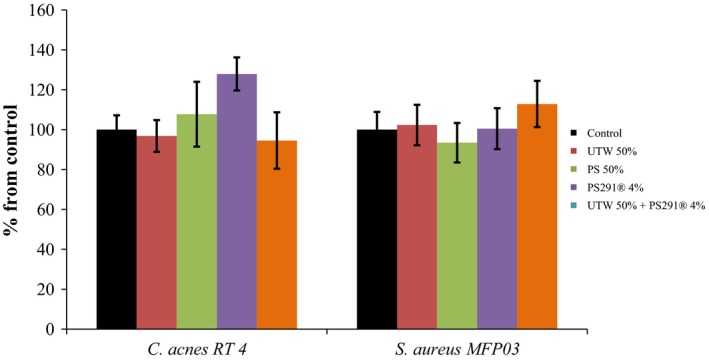
Effect of thermal water and rhamnose‐rich polysaccharide (PS291^®^) on the metabolic activity of an acneic strain of *Cutibacterium acnes* and *Staphylococcus aureus* in biofilms. The Effect of Uriage thermal water and PS291^®^ alone or in association on the metabolic activity of *C. acnes* RT4 and *S. aureus* MFP03 was measured by biofilm staining with 3‐(4, 5‐dimethyl‐2‐thiazolyl)‐2, 5‐diphenyl‐2H‐tetrazolium bromide (MTT). (a) *C. acnes* RT4. (b) *S. aureus* MFP03. All experiments were carried out in at least three replicates. Control studies were realized as indicated in Figure 1

As the labeling of crystal violet and SYTO9 Green is independent of the viability of bacteria, in order to determine the real amount of metabolically active microorganisms in the biofilm we used an MTT labeling which reveals the oxidative activity of the cells. As shown in Figure 7, PS, UTW and PS291 had limited influence on the viability of *C. acnes* and *S. aureus*. In the presence of PS291, the number of metabolically active cells increased to 119% for *C. acnes* RT4 and to 113% for *S. aureus* MFP03 but this difference was limited although significant. The association of UTW to PS291 did not modify the viability of any of the studied bacteria.

### 3.5 Effect of thermal water and PS291^®^ on the surface polarity of acneics strains of *C. acnes* and *S. aureus*

Results suggesting that UTW and PS2961 could influence the initial binding of bacteria on the surfaces, and this event being dependent of their surface properties, we used the MATS technique to investigated potential surface polarity changes.

**Figure 8:**
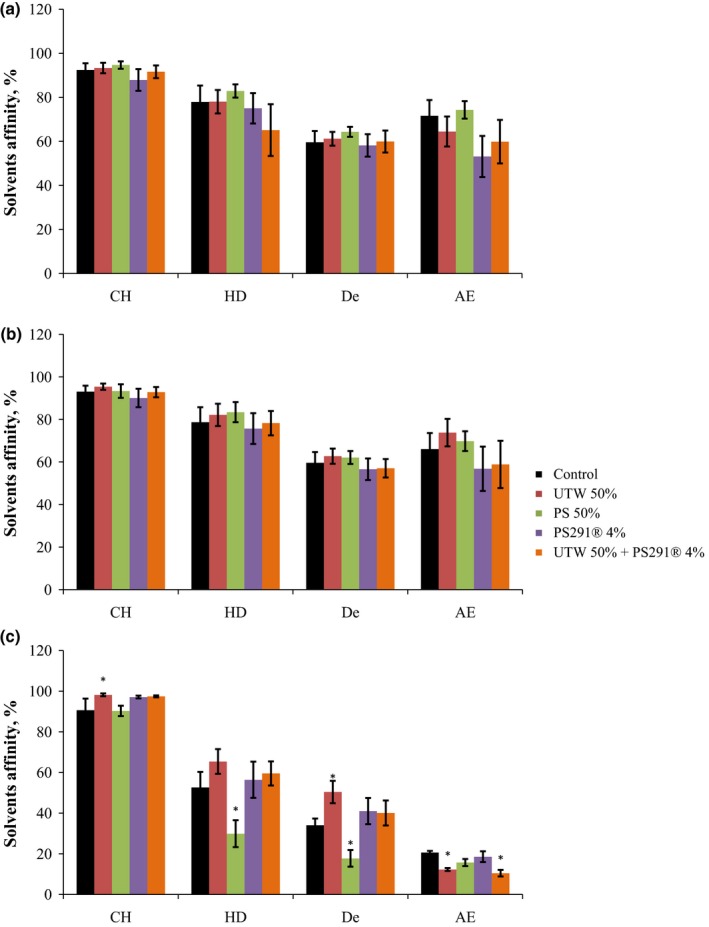
Effect of thermal water and rhamnose‐rich polysaccharide on the affinity of acneics strains of *Cutibacterium acnes* and *Staphylococcus aureus* to solvents of different polarities. (a) *C. acnes* RT4. (b) *C. acnes* RT5. (c) *S. aureus* MFP03. chloroform (CH), hexadecane (HD), decane (De) and ethyl acetate (EA). Each value represents the *M* ± *SEM* of five independent experiments. Control studies were realized as indicated in Figure 1 (*p < 0.05)

Both *C. acnes* strains exhibited high hydrophobicity and affinity to organic all solvents (Figure 8). Exposure to UTW or PS291 alone or in association was not associated to significant changes in the surface properties of *C. acnes* RT4 and RT5 strains. *S. aureus* MFP03 also showed high affinity to chloroform and more relatively to hexadecane and decane and low affinity to ethyl acetate indicating that, as *C. acnes* its surface is hydrophobic. However, the limited affinity to ethyl acetate reveals that the Lewis acid‐base properties, and subsequently the surfaces charges of *S. aureus* are not identical to that of *C. acnes*. After exposure of *S. aureus* to UTW 50% and PS291 4%, its affinity to chloroforme, hexadecane and decane increased whereas the affinity to ethyl acetate decreased. Chloroform‐hexadecane pair having almost identical Van der Waals forces, it appears that UTW and PS291 increased the hydrophobic and electronegative character of the bacterium. This is coherent with the observation of a marked reduction of the affinity of *S. aureus* to ethyl acetate. PS 50% had very different effects on *S. aureus*, particularly in regard of its affinity to chloroform, that was not modified, and hexadecane, that was markedly reduced.

## 4 DISCUSSION

Effect of active components used, or that will be used, in cosmetics industry have been studied in a number of works. This is an urgent matter in view of a global tendency to develop preservative‐free cosmetic formulae, since preservatives may cause allergy and other skin disorders (Feuilloley, Doléans‐Jordheim, & Freney, 2015; Varvaresou et al., 2009). A number of works dealt with the role of *C. acnes* in development of acne simplex and investigation of various compounds suppressing its growth or virulence. Thus, data exist on the inhibitory effect of the extract of cinnamon bark on growth of various skin microorganisms, including *C. acnes* and *S. epidermidis* (Nabavi et al., 2015), on anti‐inflammatory effect and inhibition of *C. acnes* by a complex of bakuchiol (a meroterpen isolated from *Psoralea corylifolia*), *Ginkgo biloba* extract, and mannitol (Trompezinski et al., 2016). Magnesium ascorbyl phosphate, a precursor of vitamin C, is known to suppress the processes of lipid peroxide oxidation by *C. acnes* (Lee et al., 2016). The works on *C. acnes* biofilms are however few, and no research has been carried out on the effects of cosmetic preparations on the biofilm growth by acneic forms of *C. acnes*. Similarly, only a few works dealt with the effect of cosmetic components on *S. aureus* biofilms. Extract of unripe *Juglans regia* fruits, which is used in manufacture of hair dyes, was shown to suppress the adhesion capacity and biofilm formation activity of *S. aureus* (Quave, Plano, Pantuso, & Bennett, 2008) and glycerol monolaurate, a natural surfactant used in cosmetics industry, also inhibits the growth of *S. aureus* biofilms (Hess, Henry‐Stanley, & Wells, 2014). To the best of our knowledge, this work is the first one presenting a complete study of the effects of cosmetics compounds on the adhesive and biofilm formation activity of acneic strains of *C. acnes* and of a human skin‐associated *S. aureus*.

Both acneic strains of *C. acnes* RT4 and RT5 had general similar behavior when they were exposed as planktonic cultures to UTW or PS291. However, whereas PS291 had minor effects on the growth kinetics of these bacteria, UTW was markedly reducing the lag time of the exponential growth phase. Conversely, the final biomass of RT4 was reduced whereas that of RT5 was not modified suggesting that whereas ETU can stimulate the metabolic activity of the bacteria, a lack of one or several nutriments can limit their final development. UTW was also reducing the final biomass formed by *S. aureus* MFP03 and this effect was identical to that observed using PS, UTW + PS291 or PS + PS291 indicating that the effect of UTW on the final biomass was probably due only to dilution of nutrients in the medium and that the bacteria were unable to use PS291 for their physiological requirements.

It was interesting to see that, as observed with a not characterized strain of *C. acnes* (Enault et al., 2014), UTW and PS291 showed a marked inhibitory effect on the RT4 and RT5 *C. acnes* and *S. aureus* MFP03 biofilm formation activity using the crystal violet technique. PS also decreased the biofilm formation measured by this technique but the effect was lower and the association of ETU with PS291 was more efficient on the biofilm than the association of PS with PS291. Results from confocal scanning microscopy were significantly different as PS was without effect on *C. acnes* RT4 whereas UTW and PS291 inhibited its biofilm formation. *C. acnes* RT5 and *S. aureus* MFP03 reacted to PS, UTW and PS291 as *C. acnes* RT4 strain, except that the decrease of biofilm formation was more limited. The differences observed between the two techniques should be attributed to the composition of physical properties of the surface on which the biofilms were grown as in the crystal violet technique biofilms were formed on flat‐bottomed polystyrene bottom wells whereas in confocal microscopy glass bottom wells were employed and polystyrene is considered as a hydrophobic surface whereas glass is typically hydrophilic (Dagorn et al., 2013). Concerning the *C. acnes* biofilm architecture, both UTW and PS291 affected it in a similar manner. In their presence biofilms formed by both strains were thinner, and the number of projections and structural elements on biofilm surface was reduced (this was actually only visible by direct observation). Thus, the three‐dimensional biofilm structure became simplified. It is also interesting to note that in the presence of UTW or PS291 the biofilms were more easily detached in agreement with the postulated anti‐adhesive properties of PS291 (Enault et al., 2014) and UTW (Uriage, 2018). The biofilm of *S. aureus* MFP03 formed in the presence of UTW 50% was a monolayer not differing significantly in thickness from the layer formed during initial adhesion. This effect was observed also for *C. acnes*, although it was less pronounced, possibly because of higher surface hydrophobicity.

In order to go further into the determination of the effects UTW and PS291 on the initial adhesion process of *S. aureus* we used a microcolony formation test in dynamic conditions. This test was not applicable on *C. acnes* RT4 and RT5 strains which required a culture in static and anoxic conditions. UTW and PS291 had a clear inhibitory effect on the initial adhesion step of *S.aureus* whereas PS had a limited effect. Unexpectedly, the association of UTW and PS291 only induced a partial reduction of *S. aureus* initial adhesion, of the same range observed using PS as if in regard of this process the two cosmetic compound had antagonistic effects. That should be explained by the different properties of UTW and PS291, the first one acting on the bacterial surface via its ionic charges and the second by embedding bacteria but therefore inhibiting the ionic interactions.

The absence of toxicity of UTW 50% and PS291 4% on *C. acnes* and *S. aureus* biofilms was verified using the MTT test. Except with PS291 used alone, and in a very limited range, no significant increase of metabolic activity was observed. This almost absence of effect of UTW and PS291 on the metabolic activity of both bacterial species indicates that the tested compounds are not susceptible to provoke dysbiosis in the skin microbiote. This is a requirement of the European Guideline for Cosmetic compounds 1223/2009 (Buzek & Ask, 2009). Nevertheless, MATS tests revealed that UTW and PS291 had a limited but visible effect on the surface polarity of both bacteria leading to a general increase of surface hydrophobicity and electronegative character. These properties are essential in the physical initial adhesion steps of bacteria to surfaces and these results are coherent with confocal microscope observations and microcolonies formation tests. A link between surface polarity and electronegative character and virulence has been previously demonstrated in other bacterial taxonomic groups, such as *Listeria* (Poncin‐Epaillard et al., 2013) but the sense, degree of variation, and metabolic response of bacteria in regard of surface polarity is variable between species (Poncin‐Epaillard et al., 2013).

Documenting the safety of cosmetic compounds on skin bacteria is nowadays a major industrial challenge with the economic pressure of customers for the development of preservative‐free cosmetics (Varvaresou et al., 2009) which therefore could directly affect the skin microbiote. In this regard, UE Regulation No 1223 (Buzek & Ask, 2009) requires that the absence of effect of active cosmetic compounds on the skin microbiote, or at least target microorganism, was controlled to avoid adverse skin reactions. This is particularly important in regard of bacteria such as *C. acnes* and *S. aureus*, which exist both as commensal and pathogenic strains but can also see their biofilm formation activity and virulence changing in response to modifications of the microenvironment (Feuilloley et al., 2015). The metabolic requirements of RT4 and RT5 acneic strains of *C. acnes* in regard of strict anoxic growth conditions are not allowing to study their interactions in binary biofilms with *S. aureus* and therefore the present study was realized on individual biofilms. Nevertheless, this is the first time that the physiological response of these bacteria to cosmetics was investigated and the present results suggest that UTW and PS291 are safe in regard of *C. acnes* development, although clinical tests should be required to get complete demonstration.

## AUTHOR CONTRIBUTION

A.G. performed the experiments, analyzed the data, and wrote the draft of the manuscript; V.B. provided technical assistance; L.L., A.I.N., and V.K.P. supervised the work; M.G.J.F. headed funding organization, supervised the work and assisted for manuscript writing. All authors read and approved the final manuscript.

## CONFLICT OF INTEREST

The authors have the following interests. This work was supported by public funds obtained from Evreux Porte de Normandie, Region Normandie and European Union (FEDER). Luc Lefeuvre is employed by the Dermatologic Laboratories Uriage. There are no patents, products in development of marketed products to declare. This does not alter the authors’ adherence to all policies on sharing data and materials, as detailed online in the guide for authors.
